# Vestibular Paroxysmia with Neurovascular Cross Compression and Antiepileptic Drugs: A Systematic Review and Discussion of Physiopathology

**DOI:** 10.3390/audiolres15020028

**Published:** 2025-03-12

**Authors:** Pierre Reynard, Hung Thai-Van, Alexandra Neagu, Eugen Constant Ionescu

**Affiliations:** 1 Department of Audiology and Otoneurological Explorations, Civil Hospitals of Lyon, 69003 Lyon, France; hung.thai-van@chu-lyon.fr (H.T.-V.); eugen.ionescu@chu-lyon.fr (E.C.I.); 2 Department of Physiology, Claude Bernard Lyon 1 University, 69003 Lyon, France; 3 Paris Hearing Institute, Institut Pasteur, Inserm U1120, 75015 Paris, France; 4 MS Curie Emergency Children Hospital, 077120 Bucharest, Romania; aaaneagu30@yahoo.com

**Keywords:** vestibular paroxysmia, antiepileptic drugs, cross compression syndrome, narrowed internal auditory canal, carbamazepine, oxcarbazepine

## Abstract

Vestibular paroxysmia (VP) is a disabling medical condition characterized by a high recurrence rate of vertigo. VP is classically associated with the presence of contact between a vascular structure and the cochleovestibular nerve, a neurovascular cross compression (NVCC). Antiepileptic drugs are the first-line treatment for disabling symptoms. We conducted a systematic review to evaluate their efficacy in patients with VP, and whose imaging shows NVCC. All published studies in PubMed databases until October 2024 were included. A total of seven studies were selected. Carbamazepine and oxcarbazepine are by far the most widely used drugs, but there is still a lack of data showing the efficacy of antiepileptic drugs in a large sample of patients. This suggests that a future randomized controlled trial with a satisfactory sample size of VP patients with NVCC and comparing several drugs with neuroprotective properties is needed. Further, as imaging in some of the patients with obvious clinical signs does not always support this diagnosis, we discussed VP physiopathology and the different types of VP published in the literature, including those with no radiological support for NVCC.

## 1. Introduction

Gowers in 1877 first hypothesized that the cochleovestibular nerve (CVN) could be at the origin of “auditory nerve vertigo” [1]. The vascular compression of a cranial nerve was first described in 1875 by Schultze. He reported a case of hemifacial spasm caused by an aneurysm of the left vertebral artery compressing the facial nerve [2]. Based on intraoperative observation, Jannetta et al. hypothesized in 1975 a relationship between “disabling positional vertigo” and the presence of a blood vessel compressing the CVN in the Obersteiner–Redlich zone (or root entry zone (REZ)) [3,4]. This condition was defined as neurovascular cross compression (NVCC) of the CVN. The main classic symptoms of vestibular paroxysmal (VP), as described by Brandt et al., are recurrent, stereotyped, spontaneous, and/or triggered by particular head positions or hyperventilation, characterized by brief spinning or non-spinning attacks of vertigo that generally last less than a minute. The attacks might be accompanied by hyperacusis or typewriter-like tinnitus, but the most common accompanying symptom is unsteadiness [5]. Symptoms are thought to be in relation with direct pulsatile compression by the vascular structures at the origin of ephaptic discharges or, less commonly, by conduction blocks [5]. The Bárány Society committee recommended the following diagnostic criteria for definite VP: at least 10 spontaneous vertigo attacks with or without rotation, lasting less than 1 min, stereotyped, responding to sodium channel blockers, with no other criteria for another diagnosis [6]. The incidence of VP is unknown in the general population, and in patients with vertigo, a prevalence of approximately 4% has been reported [6,7].

VP with NVCC usually refers to the presence of a vascular structure in the cerebellopontine angle (CPA) in contact with the CVN causing a localized neuropathy [5,6,8]. Adapted MRI sequences exploring the IAC with axial and coronal slices is the gold standard, allowing for the acquisition of a contact image of the CVN and eliminating other retro-cochlear pathologies in the CPA. High-resolution MRI with CISS/FIESTA sequences of the brainstem were proposed to support the diagnosis of VP by NVCC [6]. According to Haller et al., the local nerve compression seen in NVCC syndrome takes place in the transition zone, distant from the brainstem and without overlapping the REZ [7]. The distance of the affected, most vulnerable region of the myelin sheath of the CVN from the nerve’s exit zone out of the brainstem has been measured in millimeters (between 0.0 and 10.2 mm [9]). It corresponds to the long intracisternal route, which is covered by central myelin of the oligodendrocytes [10]. Furthermore, the intracisternal part of central myelin is shorter in other cranial nerves [10]. The CVN would be highly sensitive to a compression-induced dysfunction due to its long route, while only covered by central myelin [9,10]. Compression may be generated either by the anterior–inferior cerebellar artery (AICA, see for classification Mc Dermott et al. [11]), the posterior–inferior cerebellar artery (PICA), the vertebral artery, or by veins [8,9,12]. More rarely, similar clinical presentations were reported in relation to osteoma or exostosis of the IAC [13,14,15], a megalodolichobasilar artery [16], or an arachnoid cyst that stretches the CVN fibers [17]. However, the link between NVCC and VP is still debated, given that radiological criteria for NVCC can be present in non-symptomatic subjects [18], and some authors recently reported numerous VP patients without documented NVCC on MRI [18,19].

Like trigeminal neuralgia, hemifacial spasm, glossopharyngeal neuralgia, intermediate nerve neuralgia, or the myokymia of the superior oblique muscle of the orbit, the presence of a compressive vessel on the CVN may lead to a process of demyelination and/or ephaptic discharges [7]. These events could generate a pattern of vestibular and/or auditory symptoms, often of short duration, in response to a specific nerve stimulus (or trigger) [7,8]. Carbamazepine (CBZ) has been evaluated in randomized clinical trials and is considered as first-line treatment for trigeminal neuralgia. Moreover, CBZ has occasionally been reported to relieve hemifacial spasms [20].

Antiepileptic sodium channel blockers include CBZ but also oxcarbazepine (OXC), phenytoin, lamotrigine, felbamate, and sodium valproate. Voltage-gated sodium channels regulate membrane excitability, laying the foundation for a variety of physiological and neuronal processes. They are the primary targets of several widely used and investigated drug classes, including antiepileptics, antiarrhythmics, and analgesics [21]. Some antiepileptic drugs will preferentially bind to inactivated sodium channels to slow their reactivation, thus limiting repeated discharges over time. Historically, microvascular decompression was the first therapeutic option for VP patients with NVCC [3,6,22]. Currently, it is reserved for cases of VP who did not respond or do not tolerate treatment with antiepileptic drugs, and in whom the affected side can be clearly identified (risk of brainstem infarction due to vasospasm) [6]. Brandt et al. introduced the use of antiepileptic drugs as first line therapy, with low doses of CBZ and OXC (200–600 mg/day of CBZ and 300–900 mg/day of OXC) [5,23]. It was also suggested that therapeutic response should be added to the diagnostic criteria. To date, no controlled studies have been conducted on the treatment of VP. Therefore, treatment recommendations are based exclusively on limited cases and small case series, with a low level of evidence [6].

The aim of the study is, on the one hand, to review the studies using antiepileptic treatment in adult VP patients with NVCC; secondarily, the aim is to discuss the different types of VP published in literature, including those with no radiological support for NVCC.

## 2. Methods

The review was conducted according to the Preferred Reporting Items for Systematic Reviews and Meta-Analyses (PRISMA) guidelines [24]. The review was not registered on PROSPERO, and the review protocol was not prepared.

### 2.1. Search Strategy

A computerized literature search was conducted on PubMed^®^, Medline, and Cochrane platforms to identify recent, relevant publications in peer-reviewed journals. The search terms “vestibular paroxysmia” and “treatment” were combined. We included studies published until October 2024 and focused on studies using antiepileptic drugs for the management of VP with NVCC in the adult population. In line with French Health Authority recommendations, articles were ranked as 1, 2, 3, or 4 by decreasing levels of evidence.

### 2.2. Study Selection

All studies of VP with NVCC including antiepileptic treatment were selected. Studies in which antiepileptic treatment was evaluated using precisely described intervention protocols, over a short or long duration, were eligible for inclusion. Duplicates and articles written in languages other than English were excluded. Pediatric series and case reports were excluded. Studies with overlapping study populations were not excluded. All the steps in the selection and evaluation of the quality of the papers were carried out by two researchers (P.R. and E.I.), who worked independently; no automation tool was used. Cases of disagreement were referred to a third person to make the final decision (A.N.).

### 2.3. Quality Assessment

Two authors independently assessed the risk of bias (P.R. and E.I.). We used the ROBINS-I tool (r isk of b ias in n on-randomized s tudies of i nterventions) to evaluate the risk of bias. The tool consists of the following seven domains: confounding, selection of participants, classification of interventions, deviation from intended intervention, missing data, measurement of outcomes, and selection of reported results. The criteria were defined and adapted to our research question. Items were scored as low risk of bias, moderate risk of bias, serious risk of bias, or unclear based on the guidelines of the ROBINS-I tool. Consensus was reached after discussion between the two reviewers.

## 3. Results

### 3.1. Search Strategy and Study Selection

**Figure 1:**
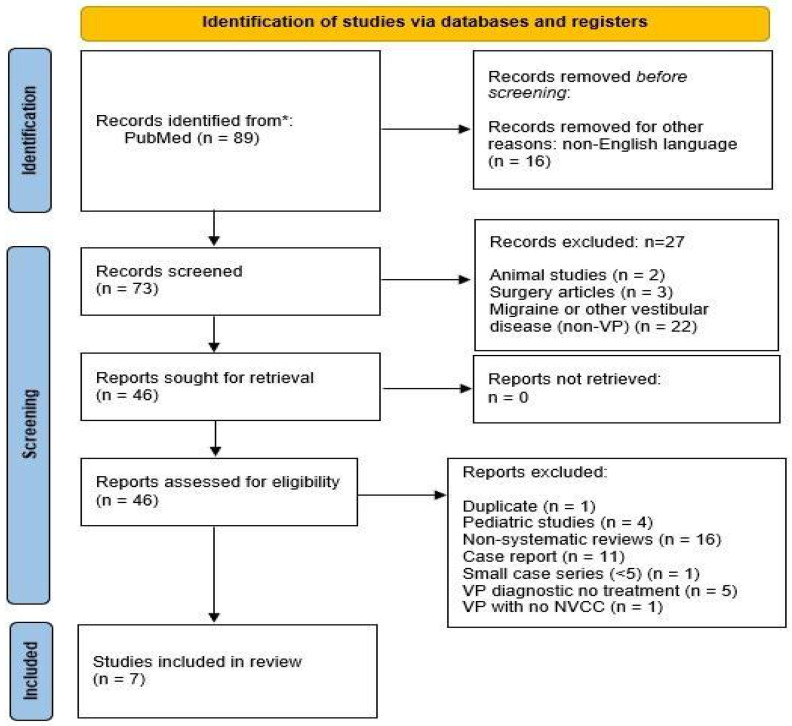
Prisma f low chart. * Cochrane database search yielded no additional articles.

A PubMed and Medline search for articles published until October 2024 using the selected keywords yielded 89 results; an additional filter was used after taking into account articles written in English, with 73 articles being selected. A review of the Cochrane database yielded no additional articles. In total, 27 articles were excluded, because they did not fall within the scope of the study: 2 articles involved animals, 22 articles focused on migraine or other vestibular pathologies, and 3 involved surgery. Finally, 46 articles were assessed. After assessing articles for eligibility, the following 39 were excluded: 5 VP studies did not focus on treatment, 1 was a duplicate, 4 concerned the pediatric population, 1 included small series (<10 patients), 11 were case reports, 16 articles were general reviews, and 1 study focused on VP without NVCC. A total of seven articles were, therefore, included for the review. The PRISMA flowchart is shown in Figure 1.

### 3.2. Data Extraction: Antiepileptic Drugs for VP and NVCC

The included studies assessing antiepileptic drugs for VP considered to be related to NVCC are presented in Table 1. The duration of the intervention (in months) was specified in all articles: 1 [25], 3 [26,27], 6 [18,19], 31 [8], or 36–48 [28]. The type of intervention (antiepileptic) was well-defined in all articles. The molecules used were OXC alone (*n* = 2) [18,25], CBZ alone (*n* = 1) [28], or a comparison of the two (*n* = 4) [8,19,26,27]. It has been suggested that betahistine mesilate (BMT), as a microcirculation improving agent, could reduce the adverse effects associated with OXC in the treatment of PV [26,27]. The sample studied varied: *n* = 18 [25], *n* = 29 [18], *n* = 32 [8], *n* = 61 [28], *n* = 146 [19], *n* = 185 [27], *n* = 196 [26].

Hüfner et al. proposed a follow-up study of 32 VP patients over a period of 31 months. A progressive and significant decrease in the attack frequency (10% of the initial value) was revealed as well as a reduction in the intensity (15%) and duration of the attacks (11%) [8]. Patients were treated with CBZ (mean dose 568 mg) or OXC (mean dose 870 mg). A total of 25 patients accepted the treatment. There was no difference between patients treated with CBZ and OXC. There were no gender-related differences noted (*p* = 0.07) or age (*p* = 0.06). The incidence of side effects in this study was not specified.

A strong association between CBZ-induced Stevens–Johnson syndrome and the HLA-B*1502 profile in Han Chinese was reported [29]. Therefore, a series of subsequent studies compared the effectiveness of OXC and CMZ to find out whether the administration of OXC alone was as effective [26,27,29]. There was evidence that betahistine mesilate tablets (BMT) as a microcirculation-improving agent could significantly improve the efficacy of CBZ in treating VP [29].

Yi et al. (2016) retrospectively evaluated the efficacy and acceptability of CBZ and OXC in combination with BMT in the treatment of VP [26]. Brandt and Dieterich’s criteria [5] were used to define VP. Patients were divided into the following three groups: CBZ alone (*n* = 73), CBZ + BMT (*n* = 65), and OXC + BMT (*n* = 58). The doses of CBZ, OXC, and BMT were 100–300 mg, 300–450 mg, and 10–20 mg, respectively, twice daily. After 12 weeks of treatment, the frequency of vertigo, the duration of vertigo, and vertigo scores (VAS) significantly decreased in all patients (*p* = 0.002), with no significant difference between the three studied groups. Thus, 32 (43.8%), 42 (64.6%), and 32 (55.2%) patients met the criteria for complete recovery in the CBZ, CBZ + BMT, and OXC + BMT groups, respectively. The difference in the response rate between the three groups was not significant. Side effects included somnolence, erythema, dry mouth, dizziness, ataxia, and nausea. The incidence of side effects was highest (30.1%) in the CBZ group, second (18.5%) in the CBZ + BMT group, and lowest (8.6%) in the OXC + BMT group. Two patients from the CBZ group withdrew because of severe side effects. The mild side effects usually disappeared spontaneously or after dose reduction. Overall, CBZ + BMT scores were highest without a significant difference [26].

In a more recent study, the same team attempted to provide new evidence with an improved study design [27]. A randomized controlled trial was conducted to assess whether CBZ and OXC had similar efficacy and acceptability in the treatment of VP and whether the improvement could be further enhanced by increasing the dose of BMT. The patients, investigators, and data analysts were blinded to the intervention methods. Half of the patients in each group (CBZ + BMT (*n* = 92) and OXC + BMT (*n* = 93)) received BMT 12 mg/time, and the other half received BMT 18 mg/time. After 12 weeks, both groups had similar average vertigo frequency, average vertigo score, average vertigo duration, and response rate. The incidence of side effects was significantly higher in the CBZ + BMT group (*p* = 0.04). Subgroup analysis found that patients receiving 18 mg of BMT had greater reductions and higher response rates than patients receiving 12 mg. The authors noted that the combination of OXC + BMT could be an alternative method for VP patients with CBZ hypersensitivity, and that the synergic effect could be enhanced by increasing the dose of BMT [27].

Although VP was described almost 40 years ago, no controlled treatment trial has been published to date. The Vestparoxy randomized, double-blind, crossover, controlled trial was proposed to provide further evidence on the efficacy of OXC (which is less neurotoxic, leading to more advantages in comparison with CBZ in these patients) [25]. The i nclusion criteria were adult patients with definite or probable VP, according to the Bárány Society ; although, radiological data were not presented. Eighteen patients were included and randomized in the study group and received OXC during the first treatment period and a corresponding placebo group during the second treatment period, or vice versa. Each treatment period lasted 3 months, with a 1-month washout period in between. The s tudied drug was administered in an ascending regimen (first week: one 300 mg tablet daily, second week: 300 mg b.i.d., third and subsequent weeks: 300 mg t.i.d.). The risk of experiencing at least one dizzy spell during the day was 0.41 with OXC and 0.62 with placebo, for a relative risk of 0.67 (*p* = 0.025). The ratio of the number of attacks on the observed days was 0.53 (*p* < 0.001) for OXC versus placebo. Median seizure duration was 4 s with OXC and 3 s with placebo. Although no serious adverse events were identified during the Vestparoxy trial, the high number of dropouts suggests that there was a substantial number of minor adverse events. The main ones cited are headache, dizziness/vertigo, fatigue, and nausea [25].

Hanskamp et al. (2022) proposed a follow-up study of patients with VP, according to Bárány clinical criteria (probable or definite), to better understand the outcome of these patients [28]. The authors considered the cause of VPs to be NVCC; although, no details regarding imaging were presented. Mean follow-up was 3.4 years. However, a small sample of patients was included retrospectively (61 patients), and an even smaller number (*n* = 12) had been treated with CBZ. Out of these, seven patients reported an improvement in frequency (*n* = 1) or in frequency and intensity (*n* = 5); four patients reported the eradication of vertigo. Interestingly, despite the lack of high-quality studies and a sufficient sample size to judge the effectiveness of the treatment for this indication, the authors reported that practitioners should be less reluctant to prescribe CBZ treatment in the presence of VP [28].

Steinmetz et al. (2022) reported a series of 73 definite VP patients treated with CBZ (200–1000 mg daily) or OXC (300–900 mg daily) [19]; 13 patients changed their initial medication during treatment due to side effects to gabapentin (*n* = 7), lacosamide (*n* = 5), or phenytoin (*n* = 1). All patients were treated for an average period of 6.5 months. The majority of patients were asymptomatic (74%) during the long-term follow-up (56% without any medication and 44% with co-medication). In the 26% remaining patients who still had symptoms, the frequency dropped significantly over the long term in 45% of the patients receiving medication and in 55% with and without medication. One-third of patients initially showed hyperventilation-induced nystagmus without specific direction.

In another interesting recent study with available radiological data, Chen et al. (2022) reported that, out of 29 patients with VP, only 65% of them had radiological criteria of NVCC on MRI, which was less than what the author had expected [18]. The response to OXC in VP patients with or without NVCC was not different (93 and 100% of patients reported an improvement within 2 and 4 weeks, respectively).

### 3.3. Quality Assessment: Efficacy of Antiepileptic Drugs in the Treatment of VP and NVCC

The critical appraisal of the studies is presented in Table 2. We identified seven studies that used antiepileptic drugs in cases of VP with confirmed NVCC on MRI. A selection bias was detected in five studies [8,10,26,27,28]. The samples of treated patients were rather limited in four out of seven studies [8,17,24,27]. Four studies [18,19,26,28] were retrospective. One study was prospective, but the used diagnostic criteria [8] were not the current Bárány criteria [6], and only 25 out of 32 patients received treatment. In addition, the doses of OXC or CBZ used in the included articles remained highly variable, even though the Bárány society [6] recalled the frequent use in the specialized literature of trial treatment for VP with low doses of CBZ (200–800 mg/day) or OXC (300–900 mg/day) [23,30].

Two studies reported significant clinical improvement in patients treated with OXC or CBZ, with no difference between the groups [26,27]; one of these included a randomized control trial and showed more adverse effects in the CBZ group [27]. However, adverse effects were only assessed in the short term. The other study included patients treated with either OXC or CBZ and showed a clinical improvement; although, this was not statistically significant and without any relevant comparison between the two subgroups [19]. Clinical improvement measurement tools were very heterogeneous. A second study was randomized, controlled, and double-blinded; however, only the OXC response was studied, and authors signaled a high drop-out rate due to adverse events [25].

Due to side effects, in some patients, the treatment was switched to gabapentin, lacosamide, pregabalin, or phenytoin [18,19]. No further data were provided for these drugs, and the sample size is far too small. To date, no other data are available in the literature for these molecules in the treatment of VPs with NVCC.

## 4. Discussion

Overall, there is still a lack of data showing the efficacy of OXC or CBZ treatment in a large sample of adult patients. This suggests that a future randomized controlled trial with a satisfactory sample size of VP patients with NVCC and comparing several drugs with neuroprotective properties is needed.

### 4.1. Physiopathology of VP

Arvanitaki in 1942 firstly introduced the term “ephapse” to designate electrical interactions between two nervous cells in contact cells, which, as a result, can generate both excitatory and inhibitory responses [31]. In the case of NVCC, nerve compression due to an ephapse-like local mechanism would result in inhibitory and activating potentials, affecting the normal frequency of the action potentials and leading to a desynchronization of the nerve activity. The presence of a blood vessel in contact with the nerve would also lead to a localized neuropathy, which may generate discharges on the nerve that can be either excitatory or inhibitory [23]. The tinnitus described by some patients would then be the result of an abnormal, distorted, or desynchronized auditory information transmitted to the auditory cortex similar to the auditory neuropathy [32].

In the study by Schwaber et al., in six patients with symptomatic CVN compression syndrome, it was suggested that the vascular loop did not play an essential role in the development of symptoms but rather acted as a trigger on an ectopically discharging nerve [33]. There was no clear evidence of demyelination in these patients; although, an axonal loss with endoneurial fibrosis was observed. The s ame findings were observed following a chronic compression or a viral infection of the nerve, such as vestibular neuronitis [33]. In some patients, incomplete nerve repair would result in a stimulating ectopic focus [34]. This local process would favor nerve stimulation and, therefore, could explain the chart of symptoms of VP. Head movements, change in body position, coughing, or sneezing would act as a “trigger” and lead to an increase in the discharge rate. It was also hypothesized that, in the case of VP, a reorganization within the vestibular nuclei would be linked either to deafferentation or to a chronic stimulation of the vestibular nerve [33].

Some authors suggested that vascular loops surrounding the internal auditory meatus do not always compress the CVN [35] and some others have pointed out that the term “conflict” would be more appropriate [36]. Although, in a recent study using 7T MRI to investigate the presence of the radiological signs of CVN in VP patients, NVCC was found in all cases, and no structural abnormalities were detected [37]. Therefore, the authors raised a number of interesting points: VP symptoms were not caused by NVCC associated with a high level of compression, as none of the patients had structural abnormalities of the CVN. Neurovascular contact would then be sufficient to induce typical VP symptoms. Further, this explains why VP-associated nystagmus is often excitatory and not lesion-induced. The absence of obvious structural nerve damage in VP also explains why, even after decades of symptoms, antiepileptic drugs can lead to complete remission of VP [37]. According to Steinmetz et al., in VP patients with no NVCC, CVN excitatory phenomena (spontaneous electrical discharges from the neuronal membranes) or central paroxysms caused by tiny brainstem lesions could occur and mimic VP [19]. A recent study used high-resolution MRI sequences to determine neurovascular compression, nerve angulation, and nerve structural integrity using diffusion tensor imaging (DTI). MRI combined with the verification of NVCC or nerve angulation and the quantification of DTI increased diagnostic accuracy at the group level but was not sufficient in a single subject (individual variability, lack of diagnostic specificity). However, NVCC was found in 10 out of 18 controls but only in 15 out of 18 VP patients. For the authors, the pathophysiology of VP could be explained by a combined peripheral and central pathology, the increased excitability of central nuclei, or dysfunction at the level of cortical or thalamic–cortical projections [38]. Brandt et al. discussed a central hyperactivity in the vestibular nuclei that could be induced and maintained by CVN compression [23].

VP pathogenesis could result in a combination of longer lasting but transient vestibular inhibition due to conduction block and paroxysmal vestibular excitations (for seconds) elicited by head movements. According to Arbusow et al., this represents a head position-dependent transition from conduction block to ectopic discharges, which have also been observed when peripheral nerves are compressed [17]. A possible change in the direction of vertigo, nystagmus, and body sway during an attack of VP makes it difficult to determine which side is affected [23]. The CVN alteration could be sequential; the progressive compression of CVN neuronal tissue in the REZ first leads to hyperactivity; subsequently, symptoms of hypoactivity and, finally, total loss of function may occur [4].

Hyperventilation Induced Nystagmus

Hyperventilation-induced nystagmus, frequently observed in case of NVCC, could be attributed to an improved axonal conduction in the vestibular nerve partially demyelinated; this effect has also been described in other diseases, such as vestibular schwannoma or in vestibular neuritis. I n the latter case, the maneuver results in a modulation of spontaneous nystagmus (inhibition, enhancement, and a reversal of spontaneous nystagmus [39,40,41]. The plane in which the eyes move during hyperventilation-evoked nystagmus may provide information about the affected semicircular canals [39]. Transcutaneously measured carbon dioxide levels have been shown to decrease after a 30 s period of hyperventilation [42]; according to Minor et al., cerebrospinal fluid pH increases in association with the reduction in pCO_2_, leading to a reduction in extracellular ionized Ca^2+^ and a transient improvement in axonal conduction in partially demyelinated nerve fibers [39]. According to Bance et al., hyperventilation can cause excitatory nystagmus and is the only test that unmasks unilateral vestibular disease without testing the dynamic properties of the vestibulo-ocular reflex [43]. Hyperventilation has been well described in VP patients with NVCC, notably in Hufner et al., where a series of 32 patients tested positive for the hyperventilation test in 70% of cases [8].

Cochlear Symptoms: The Typewriter Tinnitus

VP patients may present with various patterns of audiovestibular abnormalities [44,45]. Only a few VP patients with NVCC have been reported in the literature who only experienced cochlear symptoms, such as typewriter tinnitus. De Ridder et al. (2007) also recommended unilateral paroxysmal tinnitus as supplementary diagnostic criteria for cochleovestibular “compression” syndrome, alongside others concomitant ipsilateral symptoms, such as hemifacial spasms, otalgia, and/or hearing loss on auditory frequencies corresponding to the tinnitus pitch but also MRI confirming NVCC and abnormal auditory brainstem response (ABR), according to Moller’s criteria (see [22,46]: i.e., I-III inter-peaks interval (IPI) >2.3 ms on the affected side and/or ipsilateral peak waves II amplitude diminished by more than <30% and/or contralateral IPI III-V >2.2 ms) [36]. In the accompanying retrospective study, ABR abnormalities were correlated with the tinnitus onset and intensity. While no ABR changes could be detected in the first two years, a decrease in the amplitude of peak II and a prolongation of the I-III IPI does occur [36]. Many investigators have hypothesized on the pathophysiology of microvascular impingement disorders that were based on the demyelination of axons at the site of vascular contact. De Ridder et al. suggested that tinnitus may not be the result of such demyelination but rather due to focal changes in signal transmission [36]. Mardini first reported “ear-clicking” tinnitus responding to CBZ in patients with positive MRI criteria for NVCC of the CVN by AICA [47]. “Typewriter tinnitus” is characterized by unilateral staccato noises; however, unlike non-pulsatile subjective tinnitus, typewriter tinnitus responds very well to CBZ [45]. Moreover, its presence would be an excellent localizing sign when associated with a VP in the presence of a bilateral compression determined by imaging, the direction of the nystagmus (if any) being insufficiently informative. However, the question does not arise in practice in cases of bilateral NVCC, given that the channel blockers drugs are now first-line treatment, and their action would be effective on both sides. Sodium channel blockers bind preferentially to sodium channels in the inactivated state, to slow their reactivation, thus avoiding repeated discharges over time. This is the likely mode of action of these drugs in VP.

### 4.2. VP with No Radiologic Criteria for NVCC

Whereas some MRI studies reported 100% NVCC in VP patients, supporting the concept of a compressing vascular bundle on the CVN [44,48], some authors did not observe such a correlation in similar patients [7,19,31,49]. The series of 73 definite VP patients reported by Steinmetz et al. reported 10% of patients without NVCC criteria on MRI [19]. Chen et al. (2022) reported only 65% of NVCC on MRI in 29 VP patients, which was lower than expected for the authors [18]. The positive therapeutic response to OXC in VP supported the pathomechanism of ectopic/ephaptic discharges, regardless of whether NVCC was present or not. It has also been discussed that, when clinical presentations are consistent with VP, patients should receive this medication, even if MRI does not reveal NVCC [18] or even in case of equivocal imagery. According to Steinmetz et al., other CVN excitatory phenomena could occur and mimic VP, e.g., spontaneous electrical discharges from the membrane or central paroxysms caused by tiny brainstem lesions [19]. This hypothesis could explain certain clinical presentations that suggest the existence of a “primary vestibular neuropathy”—in the absence of any other local anatomical explanations, such as NVCC.

**Figure 2:**
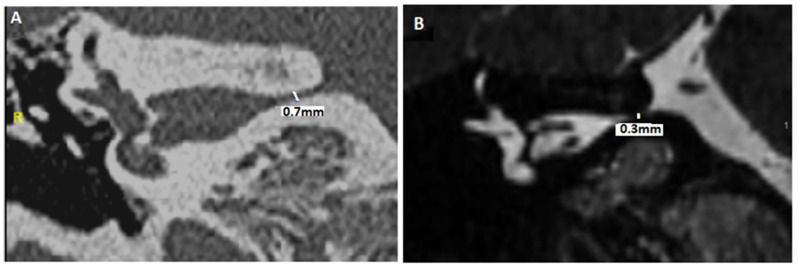
Assessment of the diameter of the internal auditory canal in the coronal plane. (**A**): HRCT of the temporal bone. (**B**): MRI, sequence: T2 (high resolution). R: right side.

**Figure 3:**
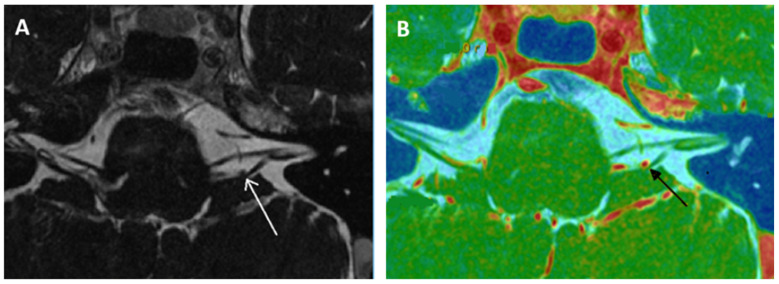
Encephalic MRI with IAC-centered slices. (**A**): T2 high resolution. Mass effect by the left posterior–inferior cerebellar artery (white arrow) on the cisternal path of the left vestibular nerve (in the root entry zone) with slight deviation. (**B**): High resolution MRI in axial plane fused image between 3DT1-weighted contrast enhanced sequence and 3DT2 DRIVE sequence. This type of sequence enables better analysis of the vascular–nervous contact (black arrow).

Recently, to explain the complaint of some quite typical VP patients in the absence of NVCC on MRI, we suggested that CVN compression secondary to a small diameter of the IAC might lead to local nerve damage, a local cranial nerve neuropathy, or even a possible ectopic excitation or inhibition of the involved cranial nerve fibers (similar or close to the NVCC) [50,51]. To differentiate it from the notion of a narrowed IAC < 2 mm with an atrophic CVN [37,52], the term near-narrowed IAC (NNIAC) was introduced [51]. Here, the compression syndrome corresponds to a conflict between the normal-sized CVN and the IAC of smaller dimensions (and, therefore, smaller volume). In the absence of a tumoral pathology in CPAs, we proposed a methodology to analyze the morphology of the container (IAC) and content (CVN) on high-resolution computed tomodensitometry (HRCT) and MRI basis in patients with VPs [51]. The novelty consisted in assessing the presence of a particular anatomical condition accompanying or not NVCC in IACs (e.g., angulation of the CVN, narrowing of the IAC in the presence of a normal-sized CVN, etc.) (Table 3). Although axial slices appeared to be less discriminating than coronal slices, it was possible to observe that near-narrowed IAC diameter cutoff values in coronal plane slices would be 3.3 mm (in HRCT) and 2.9 mm (in MRI) [51] (Figure 2). The analysis of the fusion images between high-resolution MRI T2 images and the HRCT of the temporal bones was included in order to improve the evaluation of the CVN trajectory and the detection of any deviations of its course inside the IAC in the axial or coronal planes (Figure 3).

We argued that the cochleovestibular symptoms could be related to a local entrapment-type neuropathy similar to carpal tunnel pathology or to radiculopathies caused by local compression [53,54]. Although symptoms in case of NNIAC may be similar, they appear to be more attenuated and generally less paroxysmal than in “classic” VP. Neuropathy in NVCC is secondary to an ephaptic process, generated by intermittent compression and pulsation of a vascular structure. Therefore, in NNIAC, the intimate mechanism would be a local neuropathy due to a local compression through a hard plane such as bone. We have, therefore, suggested a secondary form of VP, with less paroxysmal symptoms. It is to be noted, as we previously emphasized, that congenital IAC stenosis is usually associated with hypoplastic CVN [55], which differentiates it from NNIAC reported by our group in which the CVN is morphologically intact. Further, we reported a small cohort of 16 children who were initially suspected of benign paroxysmal vertigo but presented with VP-like symptoms. NVCC was ruled out on the basis of an MRI in half of the cases; instead, a narrowing of the IAC but with normal morphology of the CVN was suspected. In both groups, treatment with OXC was effective not only in relieving dizziness but also in normalizing electrophysiological findings, which was never reported previously [50].

We have also reported the case of a patient with an NVCC involving the AICA and the CVN within an IAC narrowed by the presence of an osteoma [15]. The p rescription of OXC provided complete symptom remission highly suggestive of secondary VP. The clinical evolution of a young patient presenting VP without NVCC and whose MRI revealed a CVN with a long pathway in a narrowed IAC, probably due to a significant hyperpneumatization of the petrous bone air cell system was also described. This patient’s symptoms were also relieved by low-dose OXC [56]. It is possible to discuss the similarity between the vertigo sometimes described by patients with arachnoidian cysts in the posterior fossa and VPs [17]. Tunes et al. (2014) had reported two patients with this localization (CPA) presenting with “vertigo and dizziness”, symptoms that disappeared after decompression [57]. The imag ing in this series indicated a compression by the arachnoid cyst on the CVN. Unfortunately, the characteristics of the vertigo were not detailed.

Symptoms in the case of vestibular schwannomas may include brief positional vertigo, typewriter tinnitus, and irritative nystagmus sometimes exacerbated by hyperventilation [40,41]. We think these symptoms should be also associated with a secondary form of VP. Treatment with antiepileptic drugs could then be proposed when surgery or radiotherapy have failed to relieve episodic vestibular symptoms, or in patients in whom these therapies are not indicated. An illustrative case report describes a 67-year-old patient with a vestibular schwannoma in the right CPA [58]. Symptoms reported by the authors were suggestive of VP and were triggered by physical activity; furthermore, a 30 s hyperventilation revealed right irritative nystagmus. Given the stability of the schwannoma and the absence of vestibular deficits, no specific surgical intervention was performed, and monitoring was decided. Although OXC (2 × 150 mg) was prescribed and well-tolerated, improvement in vertigo was not specified, but hyperventilation-induced nystagmus disappeared; however, it reappeared on the discontinuation of treatment.

Another case report of a 16-year-old child presenting with symptoms compatible with VPs and an MRI showing no NVCC on the CVN was described. An electrocardiogram showed a Wolff–Parkinson–White (WPW) syndrome, but symptoms persisted despite successful catheter ablation. The patient also had a COVID-19 infection 4 months before. Interestingly, a diagnosis of vestibular migraine was considered, but treatment with topiramate did not alleviate the patient’s symptoms; instead, a treatment with a second-line antiepileptic sodium channel blocker (CBZ) was effective. However, the type of MRI sequences (use of CISS sequences), CAI measurements, and the slices studied to rule out NVCC were not specified [59].

### 4.3. Arguments for VP Classification

We have detailed above the various studies in the literature that have more or less raised the notion of VP without an NVCC image being observed on MRI. Alongside VP, other neurovascular compressions with paroxysms have also been described on other cranial nerves [7]. Trigeminal neuralgia is the most common neurovascular compression syndrome [7], the most usual medical treatment in this case being CBZ. The third edition of the International Classification of Headache Disorders (ICHD-3) proposed by the International Headache Society’s Classification Committee proposes to classify trigeminal neuralgia on the basis of MRI findings in addition to electrophysiology. Classical forms imply neurovascular compression, secondary forms imply tumors in the cerebellopontine angle, AV-malformation, and multiple sclerosis; whereas, idiopathic forms describe trigeminal neuralgia with neither electrophysiological tests nor MRI showing significant abnormalities (https://ichd-3.org/13-painful-cranial-neuropathies-and-other-facial-pains/13-1-trigeminal-neuralgia/13-1-1-classical-trigeminal-neuralgia/13-1-1-3-idiopathic-trigeminal-neuralgia/ accessed on 1 October 2024).

Therefore, on the same basis, it is possible to propose three subtypes of VP: I—c lassic VP due to NVCC, II—secondary VP due to compression of another kind (osteoma, schwannoma, near-narrowed IAC), and III—idiopathic VP (Table 4).

### 4.4. Arguments for OXC as First Line VP Treatment?

In a study of the NVCC syndromes of cranial nerves (including VP but also superior oblique myokymia and ocular neuromyotomia), Strupp et al. proposed a brief review of the medications proposed in the literature in order to assess the suitable dosage for a therapeutic trial [30]. The authors recommended the use of low-dose CBZ (50–200 tid) or OXC (100–300 tid). If the drug was effective but not tolerated, other sodium channel blockers, such as phenytoin (100–300 mg tid), gabapentin (100–600 mg tid), or valproic acid (100–300 mg tid), were proposed; lacosamide has also been proposed in increasing doses up to 250 mg daily, alternatively, to CBZ and OXC [60]. Russel et al. suggested that gabapentin (mainly indirect calcium channel blocker) should be considered in older patients, due to CBZ’s side effects [61]. A recent radiological (7T MRI) study also used gabapentin in treating VP patients [37] with no further detail (the reason for choosing this medication was not specified). Six patients were treated with this molecule and a switch to OXC or CBZ medication was necessary in two patients due to initial therapeutic failure. There is very limited evidence in the literature for the use of topiramate, baclofen, and lamotrigin; although, these drugs have been reported as treatment options for trigeminal neuralgia [62,63].

OXC has been developed through structural variation of CBZ (ketoanalogue) with the intent of avoiding side effect -causing metabolites. It differs from the sodium current blockade of other antiepileptic drugs, such as CBZ and phenytoin, in the way that the blocking effect on the sodium channels occurs at much lower concentrations in vitro.

Unlike many other antiepileptic drugs, such as CBZ and phenytoin, OXC metabolism is not induced or inhibited (or only to a minimal extent) by the cytochrome P-450 system. Cytochrome P-450 2C19 (involved in the elimination of voriconazole and the activation of clopidogrel to its active metabolite) is only inhibited by higher doses of OXC. This may explain why there are fewer interactions with concomitantly administered drugs. However, OXCs induce c ytochromes P-450 3A4 and 3A5, which are responsible for the metabolism of oral contraceptives [64]. As a result, OXC (particularly at high doses) can lead to a loss of efficacy of oral contraceptives, and non-hormonal contraceptive measures should be used. Evidence concerning the safety of OXC during pregnancy is still insufficient [65]. In France, CBZ is considered teratogenic. Patients treated with CBZ should use an effective method of contraception during treatment and for up to two weeks after stopping it.

OXC and its metabolites are almost entirely excreted in the urine [66]. Therefore, the daily dose should be adapted depending on the renal clearance. In case of GFR <30 mL/min, the dose should be reduced, the dose escalation should be carried out more slowly, and the interval between doses should be prolonged [64,66]. Unlike OXC, CBZ is mainly metabolized in the liver, so lower daily doses of CBZ may be required in patients with hepatic diseases. The a brupt withdrawal of OXC should be avoided [67]. In general, side effects include sedation, dizziness, abnormal gait, headache, ataxia, fatigue, confusion, nausea, vomiting, abdominal pain, or rash [67] (see https://www.nlm.nih.gov/medlineplus/druginfo/meds/a682237.html for details; accessed on 1 October 2024). The risk of developing exanthems during treatment is much lower with OXC than with CBZ, and adverse effects often appeared only transiently at the start of the OXC treatment [57]. Hyponatremia and increased suicidal ideation are two life-threatening side effects in patients on OXC [67]. Hyponatremia is more likely to occur with sodium-lowering co medication [64]. In France, a report from the transparency commission of the “Haute autorité de santé” from 19 December 2001 reported that, unlike CBZ, treatment with OXC does not require systematic biological monitoring (hematological, renal, hepatic). However, since very rare cases of hepatitis have been reported, when a hepatic abnormality is suspected, liver function should be monitored and the OXC treatment discontinued (https://www.has-sante.fr/upload/docs/application/pdf/ct020841.pdf; accessed on 1 October 2024).

For all these reasons, OXC and CBZ are different antiepileptic drugs, even if their molecular structure is similar. OXC offers a number of clinically relevant advantages over CBZ and other antiepileptic drugs in terms of efficacy, tolerability, and suitability in combination with other drugs [64]. The Chinese studies presented above [26,27] and the randomized controlled trial (Vestparoxy) by Bayer et al. have recently highlighted equal efficacy advantages of OXC in comparison to CBZ [25]. There are, thus, many arguments in favor of a first therapeutic trial with OXC, rather than its historical predecessor.

## 5. Conclusions

Overall, there is still a lack of data showing the efficacy of OXC or CBZ treatment in a large sample of patients. A future randomized controlled trial with a satisfactory sample size of VP patients with NVCC and comparing several drugs with neuroprotective properties is needed. However, oxcarbazepine showed a better tolerability profile with equal efficacy to carbamazepine. As in VP, the basis of the diagnosis remains clinical, and in the case of patients without clear radiological criteria for NVCC but with evocative symptoms, a short trial treatment can be initiated on a case-by-case basis with all the necessary precautions.

Although the current clinical criteria for the diagnosis of VP appears to date as insufficiently recognized, there has recently been observed an increase in the interest for a better knowledge of this pathology. Clinicians are, therefore, encouraged to consider VP diagnosis—with or without NVCC—more often, especially in cases of stereotypical, spontaneous, or repeatable vertigo with certain maneuvers and/or positions of the body or of the head but also in the case of equivalent auditory phenomena, such as “typewriter tinnitus”. This approach will probably allow the next clinical trials that will focus on this pathology to provide clearer answers, including its actual incidence in the general population.

## Figures and Tables

**Table 1:** Articles evaluating the effectiveness of antiepileptic drugs in case of VP by NVCC. VP: vestibular paroxysmia; NVCC: neurovascular cross compression; CBZ: carbamazepine; OXC: oxcarbazepine; RCT: randomized controlled trial; DHI: dizziness handicap inventory; BMT: betahistine mesilate tablets.

Reference [X] Level of Evidence	Aim of the Study	Intervention	Results	Bias and Weaknesses
Hufner et al. (2008) [8] 4	Assessment of CBZ and OXC in VP patients	- Follow-up study of 32 patients over a mean period of 31 months - 25 patients accepted treatment (mean treatment period 25 months) - Patients treated with CBZ (mean dose 568 mg) or OXC (mean dose 870 mg)	- Significant decrease in attack frequency down to 10% - Reduction in intensity (15%) and duration of the attacks (11%) - No difference between patients - No effect of gender (*p* = 0.07) or age (*p* = 0.060). - No serious side effects occurred - Controlled double-blind trial needed	- Limited number of patients - Lack of verified diagnostic criteria at the beginning of the study - Probable and certain VP included with variable criteria
Yi et al. (2016) [26] 4	Efficacy and acceptability of CBZ and OXC in combination with BMT in the treatment of VP	- Retrospective study - 3 groups: CBZ alone (*n* = 73), CBZ + BMT (*n* = 65), OXC + BMT (*n* = 58) - Doses of CBZ: 100–300 mg, OXC: 300–450 mg, BMT: 10–20 mg, twice daily	- After 12 weeks: frequency of vertigo, duration of vertigo and vertigo scores decreased in all three groups (*p* = 0.002), no difference between the 3 groups - Difference in response rate between the 3 groups was not significant - Incidence of side effects: 30.1% for CBZ, 18.5% for CBZ + BMT, 8.6% for OXC + BMT	- No randomized controlled trial - Selection bias (same recruitment center) - Only short-term effects evaluation - Relatively small number of recruited patients (separate analysis of short and long vertigo duration to be cautiously interpreted
Xue et al. (2018) [27] 2	CBZ and OXC: similar efficacy and acceptability? - Improvement enhanced by increasing BMT dose	- RCT; double-blind - CBZ + BMT (*n* = 92) and OXC + BMT (*n* = 93) - BMT 12 mg/time, or BMT 18 mg/time	- After 12 weeks: similar vertigo frequency, score, duration, and response rate - Incidence of side effects higher in CBZ + BMT group (*p* = 0.04) - 18 mg of BMT: greater reductions, higher response rates	- Recruitment from the same site (selection bias?) - Application oOXC/CBZ and higher doses of BMT not analysed - Only short-term effects evaluation - Patients included before current VP criteria (Bárány 2016) - No studies on CBZ or OXC dose reduction with BMT (18 mg)
Bayer et al. (2018) [25] 2	To provide further evidence on the efficacy of OXC in VP patients - Patients with definite or probable VP	- RCT, double-blind, crossover - 18 patients randomized - OXC (first period) and placebo (second period), or vice versa - 1st week: 300 mg daily, 2nd: 600 mg daily, >3rd: 900 mg daily)	- Risk of at least one dizzy spell /day = 0.41 with OXC and 0.62 with placebo, relative risk of 0.67 (*p* = 0.025) - Ratio of the number of attacks was 0.53 (*p* < 0.001) for OXC versus placebo - Median seizure duration was 4 s with OXC and 3 s with placebo - No serious adverse events identified during the trial	- High drop-out rate (adverse events) - No assessment of subjective relief of symptoms or quality of life
Hanskamp et al. (2022) [28] 4	- Evaluation of outcomes in patients with VP according to Bárány clinical criteria	- Follow-up study; retrospective (61 patients) - Patients with probable or definite VP - Only 12 treated with CBZ (dosage?)	- Mean follow-up: 3.4 years. - 7 patients: improvement in frequency (*n* = 1) or in frequency and intensity (*n* = 5)	- Partly retrospective study (recall bias?) - Small sample size: only 12 patients treated - Only carbamazepine - Selection bias
Steinmetz et al. (2022) [19] 4	- To describe clinical symptoms and laboratory findings in large patient cohort of definite or probable VP - To evaluate the long-term course over years in definite VP	- Retrospective study - Series of 73 probable VP patients - Series of 73 definite VP patients treated with CBZ (200–1000 mg daily) or OXC (300–900 mg daily)	- 70%: no accompanying symptoms; 30%: mild unilateral cochlear symptoms - 13 patients changed medication (side effects) to gabapentin (*n* = 7), lacosamide (*n* = 5), or phenytoin (*n* = 1) - All: treated for an average of 6.5 months; 74% asymptomatic during long-term course (56% without medication, 44% with continuous treatment); 26% still had symptoms, frequency significantly reduced over long term (45% and 55% with and without medication)	- Selection bias due to the referral of patients to a specialized tertiary center (offset by the rigorousness of data collection) - No OXC-CBZ comparison
Chen et al. (2022) [18] 4	- To study the long-term treatment outcome of VP	- 29 VP patients - OXC (*n* = 26), pregabalin (*n* = 2), gabapentin (*n* = 1) at least 3 months (dosage? variable) - Follow-up 6 months	- Improvement with or without NVCC is not different (93/100% at 2/4 weeks) - At 8–56 months, 84.6% maintained a good response (300 and 600 mg/day). - Complete remission (no medication) >1 month (*n* = 11), with remission >12 months (*n* = 6) - 19 patients = NVCC (presence did not predict results)	- Retrospective study. - Duration of follow-up differed among patients - Recall bias (self recalled dizziness frequency) - No use of formal scales (such as DHI) - Only oxcarbazepine was used

**Table 2:** Quality assessment. Confounding: ○ non confounding (aims and variables well defined), presence of confounders, no information. Selection of participants: ○ no bias in selection, ● presence of bias, NA not applicable. Classification of intervention: ○ absence of measurement bias, ● presence of bias, Ø no information. Deviation from intended intervention: ○ absence of performance bias, ● presence of bias, Ø no information. Missing data: ○ <10% missing data, ● ≥10%, Ø no information. Measurement of outcomes: ○ similar measurement of outcomes between groups and blinding of assessors, ◐ Similar measurement and no blinding, ● difference in measurement and no blinding, NA not applicable. Selection of reported results: ○ primary outcomes reported according to the protocol, ◐ Selective report of a subgroup of participants with explanations, ● missing outcomes/data reported for a subgroup, Ø no information.

ROBINS-I Tool Risk of Bias (RoB)									
Study	Study Design	Sample Size	Bias Due to Confounding	Bias in Selection of Participants	Bias in Classification of Interventions	Deviation from Intended Intervention	Bias Due to Missing Data	Bias in Measurement of Outcomes	Bias in Selection of Reported Results
Hufner et al. [8]	PCS	32	○	●	○	Ø	○	NA	○
Yi et al. [26]	RCS	196	○	●	○	○	Ø	◐	○
Xue et al. [27]	RanCtT	185	○	●	○	○	○	○	○
Bayer et al. [25]	RanCtT	18	○	○	●	●	●	○	○
Hanskamp et al. [28]	RCS	61	●	●	●	○	●	◐	●
Steinmetz et al. [19]	RCS	146	○	●	●	Ø	Ø	◐	○
Chen et al. [18]	RCS	29	○	○	●	●	○	◐	○

**Table 3:** HRCT and MRI algorithms to assess morphology and dimensions of IACs (adapted from Ionescu et al. 2023 [51]). IAC: internal auditory canal; CVN: cochleo vestibular nerve; NVCC: neurovascular cross compression.

HRCT Diagnosis Steps	MRI Diagnosis Steps
- Evaluation of the smallest anteroposterior and craniocaudal IAC diameters (after measuring IAC length) - Description of bony abnormalities of the IAC walls (normal bone, fibrous dysplasia, meningeal calcifications, and/or osteoma of exostosis) - Evaluation of any significant angulation or deformation of the IAC (anteroposterior and craniocaudal planes)	- Assessment of the perineural fluid environment in the IAC - Angulation of the CVN? - Presence of NVCC? - Analysis of fusion images between high-resolution T2 and HRCT of the temporal bones - Evaluation of anteroposterior and craniocaudal diameters of the IAC

**Table 4:** Proposition of VP classification.

I—Classical VP	II—Secondary VP	III—Idiopathic VP
- Neurovascular cross compression (NVCC)	- Schwannoma, meningioma - Osteoma, other bony compression - Meningocele - Narrowed internal auditory canal - Arachnoid cysts of the posterior fossa	- Primary vestibular neuropathy
